# Cholesterol-to-Coprostanol Conversion by the Gut Microbiota: What We Know, Suspect, and Ignore

**DOI:** 10.3390/microorganisms9091881

**Published:** 2021-09-05

**Authors:** Catherine Juste, Philippe Gérard

**Affiliations:** AgroParisTech, Micalis Institute, Université Paris-Saclay, INRAE, 78350 Jouy-en-Josas, France; catherine.juste@inrae.fr

**Keywords:** cholesterol, coprostanol, gut microbiome, intestine, bile acids, feces

## Abstract

Every day, up to 1 g of cholesterol, composed of the unabsorbed dietary cholesterol, the biliary cholesterol secretion, and cholesterol of cells sloughed from the intestinal epithelium, enters the colon. All cholesterol arriving in the large intestine can be metabolized by the colonic bacteria. Cholesterol is mainly converted into coprostanol, a non-absorbable sterol that is excreted in the feces. Interestingly, cholesterol-to-coprostanol conversion in human populations is variable, with a majority of high converters and a minority of low or inefficient converters. Two major pathways have been proposed, one involving the direct stereospecific reduction of the Δ5 double bond direct while the indirect pathway involves the intermediate formation of 4-cholelesten-3-one and coprostanone. Despite the fact that intestinal cholesterol conversion was discovered more than a century ago, only a few cholesterol-to-coprostanol-converting bacterial strains have been isolated and characterized. Moreover, the responsible genes were mainly unknown until recently. Interestingly, cholesterol-to-coprostanol conversion is highly regulated by the diet. Finally, this gut bacterial metabolism has been linked to health and disease, and recent evidence suggests it could contribute to lower blood cholesterol and cardiovascular risks.

## 1. Introduction

**Figure 1:**
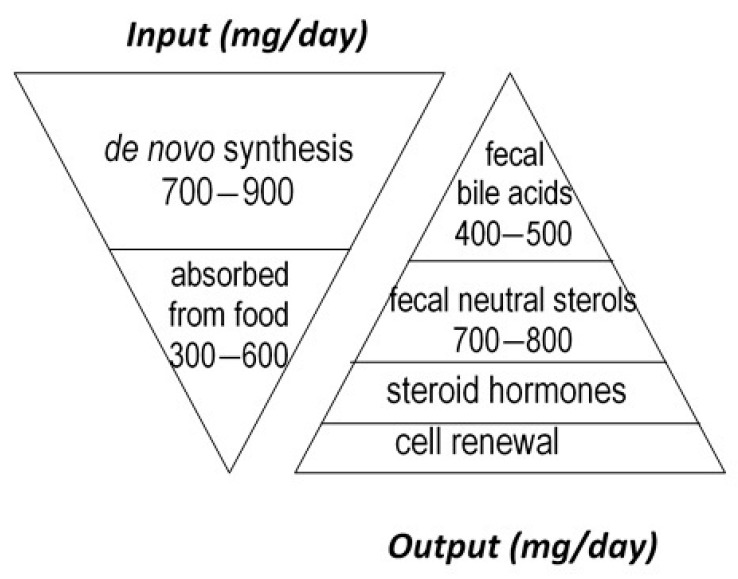
Estimation of daily cholesterol input and output in the healthy adult.

Cholesterol sustains life of most higher animals and birds as a main element of cell membrane architecture, a unique natural precursor for the synthesis of all five classes of steroid hormones (glucocorticoids, mineralocorticoids, progestins, androgens and oestrogens), vitamin D, and bile acids. Yet, an excess of cholesterol in blood is also detrimental in humans since it is recognized as a major risk of cardiovascular disease, which is a leading cause of mortality in developed countries [1]. Excess cholesterol in blood and other organs comes from an imbalance between input and output. Input originates from endogenous synthesis mainly in the liver and the small intestine, plus exogenous food intake of animal origin. Output proceeds via bioconversion to bile acids in the liver, steroid hormones in diverse tissues, mainly the adrenal cortex, testis, and ovary, cell renewal, plus enteric metabolism into virtually non-absorbable microbial derivatives, which are eliminated in the feces. Among them, non-absorbable coprostanol is by far the most predominant and of highest clinical interest for removal of cholesterol from the body. Figure 1 gives a rough estimation of daily cholesterol input and output in the healthy adult.

Considerable effort has thus been devoted to develop, question, and update reliable clinical interventional strategies aimed at lowering blood cholesterol through either depleting input, stimulating output, or both. A simple way of getting there would be to limit cholesterol input by lowering its dietary supply from animal products and/or its intestinal absorption by sequestering or competing ingredients [2], which unfortunately may not be sufficient in a number of cases where an inherited propensity to synthesize too much endogenous cholesterol limits the effectiveness of a healthy diet. For that reason, a number of medicines have been proposed to inhibit synthesis of cholesterol by the liver and intestine, the most popular and widely prescribed being statins. A complementary strategy would be to increase the use of cholesterol for bile acid synthesis. This could be done through oral intake of bile acid sequestering agents, which partially remove bile acids from the enterohepatic circulation and boost their compensatory de novo synthesis from the unique precursor cholesterol. A fourth and last strategy, which was proposed several decades ago, but failed to be put into practice up to now, would be to increase the bioconversion of cholesterol in the gut lumen to its end, non-absorbable bacterial metabolite coprostanol, which could be achieved through supplementation with either cholesterol-metabolizing microbes or microbial enzymes. The latter strategy was the matter of a series of patents and well-known papers in the field [3,4,5,6,7,8,9]. The bottleneck is, however, our limited knowledge about cholesterol metabolism by the gut microbiota, as regards to only few isolated coprostanoligenic bacterial strains and unidentified responsible genes. If cholesterol-to-coprostanol conversion has been mentioned or described in numerous previous reviews [10,11,12,13,14,15,16,17,18,19,20], the present one is the first one entirely dedicated to this gut bacterial metabolism. It constitutes an opportunity to reassess what we know or suspect about this bacterial metabolism, which, in spite of being probably as old as appearance of mammals on earth, still remains so mysterious.

## 2. Historical Evidence

The idea that gut microbes could participate in the elimination of cholesterol from the body through the bowel emerged as early as the late 19th century [21,22], 30 years after the discovery of coprostanol in human feces in 1862 [23], designated at that time as “stercorin” or “coprosterol.” Coprostanol was early ascribed to enteric metabolism since (i) it was largely recovered in stool after a healthy human had ingested cholesterol [21], (ii) it was found to be absent from meconium and feces of fasting animals [24], and (iii) it was not found in any other body compartment besides feces and intestinal contents [25]. Then, multiple evidence that the intestinal microbiota was solely responsible for the cholesterol-to-coprostanol conversion in the intestine wasbrought by a number of observations, notably (i) coprostanol was never found in feces of germ-free animals [26,27,28,29], and (ii) administration of bactericides/antibiotics abolishes/diminishes coprostanol formation in conventional animals [29,30,31] and in man [32]. Interestingly, several studies have reported that microbial cholesterol-to-coprostanol conversion in human populations was bimodal, with a majority of high converters (almost complete cholesterol conversion) and a minority of low or inefficient converters (coprostanol content representing less than one-third of the fecal neutral sterols content) [33,34].

The transformation of cholesterol to coprostanol was reproduced in vitro [35] almost one century after the discovery of coprostanol and its origin, and 20 more years were necessary to obtain the first pure culture of a cholesterol-to-coprostanol-reducing bacterium [36]. It is not for lack of interest for this unique metabolism, which finds applications in areas as diverse as medicine, biotechnologies, enzymology, environmental protection, and paleodemography, but simply because sterol metabolism is one of the most complex, and the gut anaerobes that support it are probably among the most difficult to cultivate, isolate, and preserve.

## 3. Coprostanol as a Tracer for Life on Earth and Anthropogenic Water Pollution

Coprostanol (5β-cholestanol) and 5β-phytostanols (5β-campestanol and 5β-stigmastanol) of dried feces appear to be very stable, as evidenced by gas-liquid chromatography patterns, which appear indistinguishable in more than 2000-year-old human coprolites and contemporary specimens [37]. Stanols can even persist in anoxic sediments for thousands of years [38,39,40]. Therefore, several studies have successfully used fecal 5β-stanols and their ratios to mark the presence of humans and domesticated ruminants [38,41,42]. For instance, the coprostanol:5β-stigmastanol ratio has been used to distinguish between human and ruminant fecal deposition [41] with values greater than 1.5 being considered indicative of human-sourced pollution [42]. Various other sterol indices have also been derived from sediments of archaeological sites as indicators of the agriculture practices [43,44,45,46]. The 5α-isomers of coprostanol and coprostanone, formed from the degradation of cholesterol by soil microbial communities [42], have also been proposed and used to distinguish between stanol input and preservation in a specific environment (5α isomers), and stanol input from feces (5β isomers) [47,48,49,50].

While refractory to degradation in anoxic sediments [38,39], coprostanol and other stanols degrade with considerable rates in effluent and sea water [39,51]. Thus, fecal stanols are suitable markers for sewage dispersion in coastal waters where the relatively shallow waters and slow current do not allow sewage particles to stay in suspension in the water column. Once incorporated into anoxic sediments, stanols are expected to persist, and any decline in concentration can be attributed to physical sediment transport [39]. Thus, metrics used to trace sewage contaminants in sediment or sewage sludge dispersion are the same as those used for sediments of archaeological sites [52,53].

## 4. Gut Microbial Metabolites of Cholesterol and Suspected Pathways

**Figure 2:**
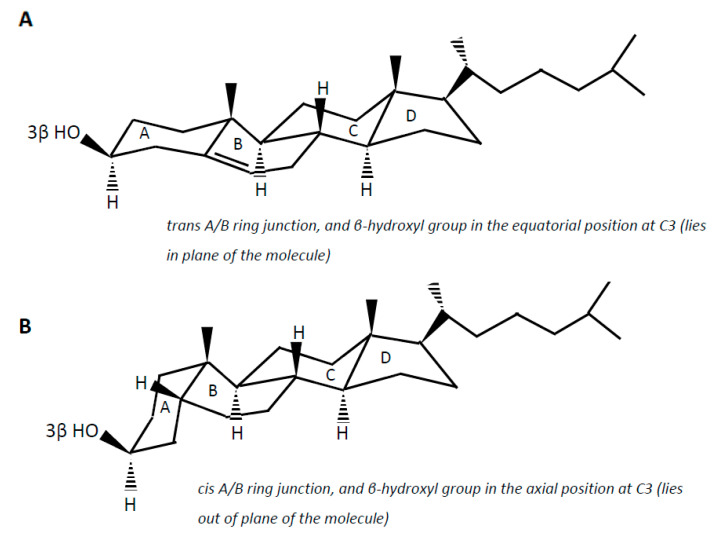
Planar representation of cholesterol (**A**) and coprostanol (**B**).

Cholesterol (examples of synonyms 3β-hydroxy-5-cholestene or 5-cholesten-3β-ol) in the intestine may be either absorbed or undergo microbial conversion to different metabolites, of which non-absorbable coprostanol (examples of synonyms 5β-cholestanol or 5β-cholestan-3β-ol) is the end and predominant product found in feces. Note that multiple synonyms can be employed for a same steroid molecule, which does not help the reader to find his way. Cholesterol is a 27-carbon molecule with a structure formed by a polycyclic ring skeleton with a trans A/B ring junction, a β-hydroxyl group in the equatorial position at C3 (i.e., in plane of the molecule), a double bond at C5 (Δ^5^ double bond), two methyl groups at C10 and C13, and a side chain at C17 (Figure 2A). Cholesterol metabolism by gut bacteria seems to be limited to the end product coprostanol and its intermediates, with no degradation of the side chain, and no fission of the steroid rings, which, in aerobic soil bacteria, lead to the complete degradation of the steroid molecule up to CO_2_ [54,55]. This could be interpreted as a symbiotic relationship between the gut cholesterol-metabolizing microbiota and the host, which participates in the elimination of excess cholesterol from the body without altering the integrity of the intestinal cell membranes. On the other hand, the significance of cholesterol-to-coprostanol reduction to the physiology of the bacteria remains to be elucidated. Eyssen et al. [56] speculated that cholesterol acted as a terminal electron acceptor in cholesterol-reducing bacteria, thereby supplying energy by means of electron transport. However, several active strains do not require cholesterol for growth, indicating that some cholesterol reducing microorganisms can use alternate electron acceptors, or generate energy by other ways [57].

Coprostanol is the saturated analogue of cholesterol. In addition to the saturation of the Δ^5^ double bond, the structure of coprostanol differs from that of cholesterol by a *cis* A/B ring junction, which causes the A ring to be bent into a second plane at approximately a right angle to the B:C:D rings, and the 3-hydroxy group to be in the axial position, i.e., out of plane of the molecule (Figure 2B). Such a structure would be mainly responsible for the poor ability of coprostanol to be esterified within the intestinal mucosa, and therefore to be absorbed [58,59,60].

**Figure 3:**
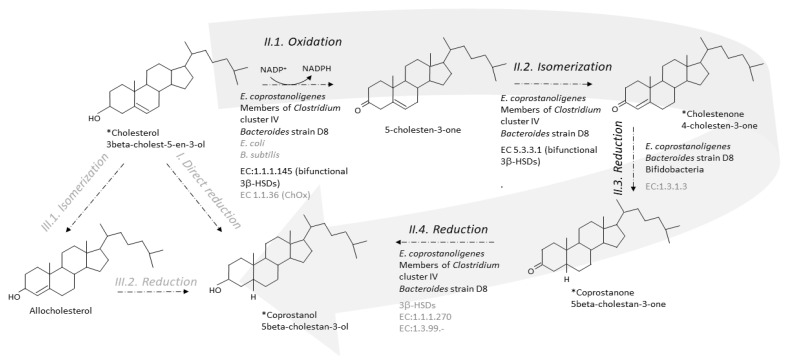
Direct and indirect pathways for the conversion of cholesterol to coprostanol by the gut microbiota. The indirect pathway (II, large grey arrow) is by far the most probable as evidenced by identification of the intermediates in crude feces and cultures of pure active strains. The direct pathway (I) cannot be excluded but never received any proof of evidence. Note that isomerization of cholesterol to allocholesterol and then reduction of allocholesterol to coprostanol (pathway III) are chemical reactions that, until proved otherwise, do not proceed in any organism. * Intermediate seen in GC-MS analyses of feces or pure active cultures. Micro-organisms and enzymes involved in the considered reaction are mentioned; black and grey characters correspond to confirmed and uncertain knowledge, respectively. A micro-organism is confirmed if it has been shown to process to the reaction in pure culture. An enzyme is confirmed if it has been purified and characterized in at least one gut micro-organism.

Two metabolic pathways have been proposed to explain the conversion of cholesterol to coprostanol by intestinal microorganisms [35,61]. One pathway is the direct stereospecific reduction of the Δ^5^ double bond [35] (Figure 3). The other pathway involves the intermediate formation of Δ^4^-cholelesten-3-one (or 4-cholelesten-3-one, or cholestenone) and coprostanone [61] (Figure 3). A series of complex in vivo and in vitro experiments using labeled and double-labeled steroid molecules was conducted over almost two decades, to follow the outcome of the hydrogen atom of the 3-hydroxy group [35,61,62,63] and prove the formation of the intermediates 4-cholesten-3-one and coprostanone [61,64]. This led to the incontrovertible evidence that conversion of cholesterol to coprostanol proceeds at least in part, if not largely, via the intermediate-involving pathway, while participation of a direct pathway is not proved but cannot be excluded [61]. Since then, many patterns of neutral fecal sterols by gas liquid chromatography, coupled or not with mass spectrometry, have referred to the presence of coprostanol, 4-cholesten-3-one and coprostanone in feces from humans and conventional animals, while all three metabolites are totally absent in germ-free models [26,27,28,29].

## 5. Isolated Active Strains

As intestinal conversion of cholesterol to coprostanol is a metabolism specific to the anaerobic intestinal environment, it was naturally assumed to be carried out by strict anaerobes, and active microorganisms were searched and isolated from anaerobic ecosystems, namely, feces of animals and humans, or sewage sludge. In fact, consistent work but modest results clearly illustrate the challenge.

In 1973, Crowther’s group [65] claimed that several pure cultures of intestinal bacterial strains, including *Clostridium*, *Bacteroides*, and *Bifidobacterium*, converted cholesterol to coprostanol when grown in the brain-based medium of Snog-Kjaer et al. [66]. Some strains of *Bacteroides* were reported to convert more than 50% of the cholesterol substrate after 7 d of incubation [65]. Since the active strains had been lost, Sadzikowski et al. [67] tried to repeat the experiment with the same methods and media, but they failed to isolate any cholesterol-reducing bacterium. The inability of subsequent investigators to demonstrate cholesterol reduction by pure cultures of similar strains remains unexplained.

The first isolation of a pure culture of a cholesterol-reducing bacterium was most likely accomplished by Eyssen et al. [36], with *Eubacterium* sp. strain ATCC 21408 isolated from rat cecal contents. In addition to cholesterol, *Eubacterium* 21,408 also reduced 4-cholesten-3-one to coprostanol. Sitosterol, campesterol, stigmosterol, 5-androsten-3β-ol-17-one, and 5-pregnen-3,20β-diol were also converted to their corresponding 5-hydrogenated derivatives. During the same period, the same group [68] showed that cholesterol was extensively hydrogenated into coprostanol in conventional rats, or gnotobiotic rats associated with a reducing *Clostridium* species plus *Eubacterium* 21,408. However, production of coprostanol was abolished within 2 days after cecotomy, and *Eubacterium* 21,408 was unable to develop in the intestine of cecectomized rats. As disappearance of the bacterium could not be related to modification of the pH or *E*_h_ values of the colonic contents, it was attributed to the fact that the cecum was necessary to maintain “normal microecology” of in this animal model.

Then, another active strain, *Eubacterium* 403, was isolated from baboon feces [69]. Both isolates, *E.* 21,408 and *E.* 403, grew for only a limited number of transfers in brain-free media supplemented with cholesterol dispersed with lecithin. For long-term maintenance, the addition of homogenized brain or lipid extracts of brain was required. By separating an organic solvent extract of brain into its components, it was determined that the bacteria required plasmenylethanolamine (PLE) for growth [69]. When pure PLE was added to a medium that contained cholesterol dispersed with lecithin, the Eubacteria grew, formed fibers, and solidified the medium. The cultures also catabolized PLE, which disappeared with cell growth.

Then, nine new active strains were isolated from dilutions of feces or intestinal contents of baboons plated on cholesterol-brain agar (CBA) [57]. Among them, two could reduce cholesterol in the absence of PLE. Unfortunately, all these strains were lost, probably due to their challenging cultivability.

Later, an easier-to-culture strain, *Eubacterium* sp. strain HL, ATCC 51,222, known as *Eubacterium coprostanoligenes*, was isolated by plating hog sewage lagoon onto a modified lecithin agar medium [70]. This isolate was claimed to have characteristics that make it a better candidate for future applications, compared to previous isolates, including the ability to grow without cholesterol or PLE, aerotolerance, and the ability to form colonies on a solid medium simply incubated in GasPak jars.

As the first two active microorganisms isolated by independent groups from different environments (*Eubacterium* sp. strain ATCC 21408 from rat feces [36] and *E.* ATCC 51222 from sewage sludge [70]), were identified as members of the genus *Eubacterium*, it was extrapolated that active strains should mainly belong to this genus [32]. Yet, intriguingly, the two microorganisms essentially differed in their growth requirements, the former but not the latter having an absolute requirement for large amounts of cholesterol or other related Δ^5^-sterols. The large amount of steroid required for maximal growth (no less than 1.5-2 mg/mL broth) led to speculate that cholesterol did not act as a growth factor but that the Δ^5^-bond of cholesterol and plant sterols is a hydrogen acceptor in the microorganism’s metabolism [36]. 

The most recently isolated and only strain from human origin is *Bacteroides* sp. strain D8 [71], isolated on CBA in 2007, from feces of a man previously identified as a high cholesterol-to-coprostanol converter, based on gas chromatography pattern of his fecal neutral sterols. Phylogenetic tree construction showed that this strain clustered in an independent clade with two isolates of the *Bacteroides dorei* species. Nevertheless, no cholesterol-reducing activity was detected in *B. dorei* type strain cultures. As observed with *E. coprostanoligenes*, *Bacteroides* sp. strain D8 started to reduce cholesterol to coprostanol on the third day of growth in vitro, and seven days were necessary to achieve complete cholesterol conversion. The intermediate products, 4-cholesten-3-one and coprostanone, were detected during cholesterol conversion by *Bacteroides* sp. strain D8, and this strain was able to convert 4-cholesten-3-one and coprostanone to coprostanol in vitro, indicating an indirect pathway for coprostanol production. The cholesterol-to-coprostanol reduction efficiency of resting cells of *Bacteroides* sp. strain D8 was found to be 0.57 mg (1.5 μmol) cholesterol reduced/mg bacterial protein/h [71], which is higher than the maximum yields previously obtained with *E. coprostanoligenes* ATCC 51,222.

In the early 2000s, a number of non-intestinal, safe, class 1 bacteria were tested for their ability to reduce cholesterol in an attempt to use them as new probiotics. Unfortunately, none of sixteen probiotic strains belonging to the genera *Bifidobacterium*, *Enterococcus*, *Lactobacillus,* and *Streptococcus*, was found to be able to convert cholesterol to coprostanol in vitro in a test medium composed of peptone yeast extract enriched with freeze-dried calf brain, or in vivo in GF mice mono-associated with a probiotic strain [72]. A few years later was the attractive discovery of five active strains of *Lactobacillus* [73]: two strains of *L. acidophilus* (ATCC 314, FTCC 0291), two strains of *L. bulgaricus* (FTCC 0411, FTDC 1311)*,* and one strain of *L. casei* (ATCC 393). When cultivated in cholesterol-enriched medium, these strains were shown to incorporate cholesterol into the phospholipid bilayer of their cellular membrane, and to reduce a fraction of it to coprostanol, as determined by fluorometric assay for cholesterol reductase activity in culture supernatants and harvested lysed cells, as well as HPLC quantification of both cholesterol and coprostanol in the culture supernatant. Surprisingly, no further action has been taken since this seemingly promising discovery.

As only a few active strains could be isolated, it has been postulated that microbial conversion of cholesterol to coprostanol would be carried out by only a few microbes indigenous to the human microbiota [32]. Yet, we evaluated the most probable number (MPN) of active bacteria, which fell between 10^7^ and 10^9^ active bacteria per g of stools in high cholesterol converters [34]. Similarly, an impressive large-scale multiomics study [74] showed that metagenomics species (MSPs) encoding 3β-hydroxysteroid dehydrogenase (HSD) (an enzyme involved in the first step of the reduction of cholesterol to coprostanol, see paragraphs below) are frequent and abundant in a large proportion of human microbiomes from diverse geographic locations. Thus, the poor availability of isolated active strains rather means that the activity is carried out by a number of sensitive anaerobes whose isolation, culture, and preservation are challenging.

The information about these isolated active strains is summarized in Table 1.

## 6. Step-by-Step Conversion of Cholesterol to Coprostanol

Evidence of cholesterol reduction via the indirect pathway being established, efforts have been made to characterize each step involved, and to identify and purify the corresponding enzymes. Here is where it gets tricky, as limited progress has been made since the isolation of the first active strains.

### 6.1. From Cholesterol to 4-Cholesten-3-One

The first step of the indirect pathway is the oxidation of cholesterol to 4-cholesten-3-one. Two types of known enzymes are able to catalyse this reaction, cholesterol oxidase (ChOx, EC 1.1.36) and 3β-hydroxy-Δ^5^-steroid dehydrogenase (3β-hydroxy-Δ^5^-HSD, EC 1.1.1.145) [75]. Both are bifunctional enzymes, which carry out two separate, sequential reactions. The enzymes first oxidize the 3β-hydroxy group of cholesterol into the 3-keto group of 5-cholesten-3-one, and then isomerize the double bond in the steroid ring backbone, from Δ5 to Δ4, giving 4-cholesten-3-one (EC 5.3.3.1, steroid Delta-isomerase) (Figure 3).

Importantly, ChOx are oxygen-dependent enzymes, and they are found in hundreds of various aerobic cholesterol-degrading soil microorganisms, including bacteria, molds, and yeasts [76,77]. ChOx is the first enzyme in the oxic pathway of cholesterol mineralization to carbon dioxide [78]. To our knowledge, only two inhabitants of the gastrointestinal tract, both facultative anaerobes, namely, one *Escherichia coli* strain isolated from the feces of a colon-cancer patient [79], and one *Bacillus subtilis* strain isolated from fecal tiger excreta [80], were reported to support ChOx activity in aerobic cultures. We also found genes that share 30–60% sequence identity with ChOx in the genome of *Bacteroides* strain D8 (unpublished data). Although it is unlikely that ChOx could participate in the conversion of cholesterol to 4-cholesten-3-one in the anaerobic environment of the lumen, it cannot be ruled out that the mucosa-associated microbiota could oxidize cholesterol in an oxygen-dependent enzymatic reaction. No homologs of any queried ChOx could be found in the genome of *E. coprostanoligenes* or among almost six million non-redundant complete genes assembled from the metagenomic sequencing of more than three thousand human fecal samples from various countries of the world [74]. In contrast, within the same study [74], a 3β-HSD was found in *E. coprostanoligenes* ATCC 5122. The enzyme, named IsmA for “Intestinal steroid metabolism A,” was overexpressed in *E. coli*, purified using HisPur™ Ni-NTA Resin, and partially characterized [74]. It is an NADP+ dependent, oxygen independent, and cholesterol inducible HSD that oxidizes cholesterol to 4-cholesten-3-one and coprostanol to coprostanone, but does not accept the 3α-hydroxy bile acids cholic and chenodeoxycholic acids as substrates. Based on massive amount of correlations combining huge metagenomics and metabolomics datasets from several cohorts in the world, six homologs of IsmA were also tracked from uncultivated members of the human gut microbiota [74]. They were overexpressed in *E. coli*, and all six lysates were confirmed to oxidize cholesterol to 4-cholesten-3-one as well as coprostanol to coprostanone. When binning co-abundant genes into metagenomic species (MSPs) across more than 3000 metagenomics datasets, 20 different MSPs containing *ismA* genes were identified. They formed a coherent clade in the phylogenetic neighbourhood of *Clostridium* cluster IV, which contains species linked to host health, including short-chain fatty acid producers. IsmA-encoding MSPs had a high relative abundance (average 1.4%) in human metagenomes, and the percentage of metagenomes containing at least one IsmA-encoding MSP varied from 37% to 92%, which supports the idea that IsmA-encoding bacteria are prevalent constituents of the human gut microbiome [74]. However, they do not exclude implication of other enzymes, since functional predictions based on sequence similarity may be difficult in the particular case of enzymes where the actual enzymatic reactions or substrates may differ even at high sequence similarity, and vice versa [81,82].

### 6.2. From 4-Cholesten-3-One (Cholestenone) to 5β-Cholestan-3-One (Coprostanone)

In 1973, Björkhem and coworkers [64] obtained a crude enzyme preparation from supernatants of freeze-pressed cecal contents of rats, which, when incubated with [4-^14^C]cholesten-3-one, produced coprostanone as the only product. The 3-oxo-Δ^4^-steroid 5β-reductase activity required NADH as cofactor. The semi-purified enzyme preparation also catalyzed the reduction of the Δ^4^-double bond of progesterone and testosterone but not the Δ^5^-double bond of cholesterol, pregnenolone, or dehydroepiandrosterone. The mechanism of reduction of Δ^4^-double bonds in 3-oxo-Δ^4^-steroids by the microbial 3-oxo-Δ^4^-steroid 5β-reductase was found to involve transfer of hydrogen from the 4*B*-position of NADH to the 5β-position of the steroid. We could not find any recent proof of 3-oxo-Δ^4^-steroid 5β-reductase activity in the gut microbiota, and no bacterial 3-oxo-Δ^4^-steroid 5β-reductase; EC:1.3.1.3, has never been purified nor can be found in the databases (Figure 3). However, three genes predicted to encode cholestenone 5β-reductase, EC 1.3.1.3, were found to be upregulated when *B. bifidum* PRL2010 was grown in presence of cholesterol [83] (Figure 3). One of them had a sequence that was found to be highly conserved (identity greater than 80 % and coverage 100 %) in the genomes of ten other bifidobacteria, all isolated from the mammalian gut [83]. When a large daily inoculum is administered (10^9^ cells per day of overnight cultures over 20 days) to the hypercholesterolemic apoE-KO mice, *B. bifidum* PRL2010 tended to lower plasma cholesterol in this small-sized experiment (5 mice treated and 5 untreated). Genes sharing 30–60% sequence identity with cholestenone 5β-reductase, EC 1.3.1.3, were also found in the genome of *Bacteroides* strain D8 (unpublished data).

### 6.3. From Coprostanone to Coprostanol

Cultures of *E. coprostanoligenes* [84] *and* *Bacteroides* sp. strain D8 [71], efficiently convert coprostanone to coprostanol, but the responsible enzyme (EC:1.1.1.270 or other 3-keto reducing enzymes) has never been characterized (Figure 3). However, it was recently suggested that 3β-HSDs could participate in this last step of cholesterol-to-coprostanol conversion by the human gut microbiota [74].

## 7. From Birth to Elderly

Although data exist regarding gut colonization before birth, it is widely accepted that our gut starts accumulating microbes during and after birth. Infants and babies have low gut microbial diversity. Our gut microbiota becomes more complex, diversified, and stable as we become adults. As we move into old age, this stability tends to fall and the composition of our gut microbial communities again varies [85]. 

That meconium of new-born infants does not contain coprostanol (formerly “stercorin”) was reported from the late nineteenth century [24], and this was ascribed to lack of bacterial “fermentation” in the foetal intestine. This is in line with the combined knowledge acquired since then, that the foetus is free from germs in the uterus until the rupture of the foetal membranes, and that colonization of the digestive tract evolves over the first years of life to reach equilibrium and stability around 3 y of age [86,87,88]. In parallel, it was repeatedly confirmed that coprostanol and intermediate microbial metabolites of cholesterol are found at very low or even undetectable levels in stools of children under 12–18 months of age [89,90,91,92,93,94]. Their levels progressively increase with advance in age to reach patterns approaching those observed in adults, in children aged 3 to 4 [89,90,91,92]. Importantly, though not surprising, a strong correlation was found between intensity of microbial metabolism of cholesterol to coprostanol and that of primary to secondary bile acids from birth until adulthood [89,90]. The initiation of microbial cholesterol metabolism would be somewhat delayed in breast-fed compared to bottle-fed children [93], but this would not preclude of the efficacy of later cholesterol biotransformation, since both breast- and bottle-fed children can become either low or high cholesterol-to-coprostanol converters at 2 y of age [93]. Partitioning of the population into low and high cholesterol metabolizing microbiota has been confirmed in youth, young adulthood [94] and adulthood, low converters (coprostanol/cholesterol + coprostanol fecal ratio <0.5) representing about 5–30% of adults from the USA [33,95], South Africa [96], and Europe [32,34,95,97,98]. However, it is not clear whether this trait is stable or not over an individual’s life, or dependent on changes in diet or any other endogenous or exogenous factor, as no longitudinal study over decades of life is available. A reduction in the number of low- and non-converters has been observed in the older age categories of a large cohort of European healthy adult men (*n* 286, 18–81 years of age), but not in women (*n* 347, same age interval) [32,99]. The authors hypothesized that a lower capacity to convert intestinal cholesterol to coprostanol could be associated with a higher risk of leaving the healthy fraction of the population prematurely.

Factors affecting efficacy of cholesterol conversion may be the composition of the intestinal microbiota, antagonistic bacteria, the physical state of the cholesterol molecule in the intestine, or the intestinal transit time [33]. The high and low conversion patterns were found to be equally distributed with respect to sex and independent of age [33]. Moreover, the efficiency of cholesterol conversion in the human gut as measured by the ratio of coprostanol to cholesterol in stools was shown to be related to the abundance of cholesterol-reducing bacteria estimated by the Most Probable Number method [34]. That the “low” or “high” phenotype can be transmitted to germ-free born rats colonized with the microbiota of either a low- or high- converter human donor would designate the microbiota structure and functions as a main driving force [100]. But we do not know which particular structural and functional features of the microbiome accompany these different profiles. A study looked for an association between the gut microbiota composition and the amount of coprostanol in the stools of seven patients treated with antibiotics for a *Clostridium difficile* infection, and six healthy volunteers with or without a recent exposure to antibiotics [101]. In this study, low levels of coprostanol were observed in stools of all patients but one, and coincided with low relative abundances of 63 taxonomic units belonging to the families *Lachnospiraceae* and *Ruminococcaceae* (order *Clostridiales*), which, in an independent study, also correlated with high levels of HDL-cholesterol [102]. In the large-scale study of Kenny et al [74], IsmA-encoding MSPs that best correlated with coprostanol level in stools, were close phylogenetic neighbours of marker species for Clostridium cluster IV and cluster XIVa, which does not exclude other MSPs that would encode coprostanol-related functions not interrogated in this study.

## 8. Dietary Regulation

Any dietary strategy aimed at decreasing cholesterol absorption in the proximal intestine and increasing cholesterol-to-coprostanol biotransformation in the hindgut would be beneficial for blood cholesterol lowering, provided that the coprostanol producing phenotype is present. Many studies report the results of dietary strategies to lower blood cholesterol in man or diverse animal models. However, few of them include a profile of fecal sterol excretion among which we can gather little significant information on cholesterol-to-coprostanol transformation and excretion rates. Importantly, the purpose of this paragraph is not to summarize the extensively documented dietary regulation of blood cholesterol, but to bring together what we know about the effect of the diet on biotransformation of cholesterol in the gut. Some effects of dietary sugars, fatty acids, proteins, and amino acids, as well as specific diets, are reported below.

### 8.1. Sugars

In rats fed a lactose-supplemented diet, intestinal absorption of dietary cholesterol markedly increased, and formation of coprostanol in the hindgut decreased, compared to a sucrose-based diet. In parallel, total liver cholesterol increased, but these effects were counteracted when the lactose diet was supplemented with calcium chloride [103]. The authors postulated that calcium formed insoluble complexes with phospholipids or fatty acids, which inhibited intestinal cholesterol absorption. In another study, diverse sugars such as lactose, galactose, fructose, sucrose, or glucose decreased the efficiency of conversion of radiolabeled cholesterol to coprostanol by human fecal homogenates in vitro, and this was attributed to a shift down in the pH [104]. Therefore, both availability of cholesterol and physiochemical conditions in the hindgut would impact the efficiency of cholesterol to coprostanol conversion.

### 8.2. Fatty Acids

Marked differences in neutral sterol excretion were reported when rats were fed either linoleic, oleic, or palmitic acid, as well as a fat-free diet [105]. The most striking difference among groups was in the partition of the coprostanol-to-cholesterol ratio in the feces. Linoleic acid markedly increased the reduction of cholesterol to coprostanol, so that the latter became the major neutral sterol present in the feces. Conversely, diets containing either oleic or palmitic acids decreased coprostanol formation [105]. Interestingly, the essential omega-6 polyunsaturated fatty acid, linoleic acid, is now recognized to decrease cardiovascular lipid risk markers in healthy individuals [106], but to our knowledge, the relationship with the microbial metabolism of cholesterol has never been addressed.

### 8.3. Protein Source and Amino Acids

In different animal models, our group [107] and others [108,109,110] demonstrated that microbial metabolism of acidic and neutral steroids was stimulated by soy protein compared to casein. In parallel, soy protein, in full or partial substitution for casein, has a well-known hypocholestrolemic effect, which is suppressed if methionine is added to the soy diet, naturally deficient in this amino acid [111]. Coincident with this, a positive correlation has also been repeatedly found between blood cholesterol and the dietary methionine supply or the dietary methionine:glycine ratio [112], and the addition of glycine to methionine-enriched diets suppresses the hypercholesterolemic effect of methionine [113]. More recently, it was discovered that a low dietary methionine:glycine ratio also coincided with a cardioprotective low homocysteinemia [114,115]. While these effects are currently investigated from the perspective of host metabolism, implication of the microbiome partner should absolutely be considered in the future. Indeed, when rats colonized by a human microbiota from a high converter donor were fed a “humanized” diet added with 3% methionine, conversion of cholesterol to coprostanol was completely abolished, and this was rapidly reversed as soon as methionine was withdrawn (personal observation).

### 8.4. Vegetarian Diets and Plant Sterols

Fecal concentration of cholesterol and its microbial metabolites, either expressed per g of dry [116] or wet [117] feces, is generally reported to be highest in omnivores, lowest in vegans, and intermediate in lacto-ovovegetarians, but total excretion of neutral sterols per 24 h would be higher in vegetarians [116]. Interestingly, mean efficiency of microbial cholesterol metabolism, expressed as the ratio of cholesterol to its microbial products, would not essentially differ between dietary habits [116,117], and the same percentage of low converters (around 20%) would be found in omnivores and vegetarians [116]. In a way similar to cholesterol, plant sterols are metabolized by the gut microbiota. In this respect, main bacterial metabolites are ethylcoprostanone, ethylcoprostanol, and sitostanol for β-sitosterol, methylcoprostanone and campestanol for campesterol, and stigmastenol and ethylcoprostenol for stigmasterol [118]. Independent studies agree that cholesterol metabolism by the gut microbiota is less efficient when high supraphysiological doses of plant sterols are consumed daily by healthy human volunteers [118,119], suggesting that the gut microbiota could preferably use plant sterols as substrates when present in greater proportions than cholesterol [118]. The same was observed in hamsters fed a hypercholesterolemic diet [120], providing evidence that the higher plant sterol consumption, the lesser microbial cholesterol metabolism. At the higher dose (0.2% by weight of the diet), plant sterols were effective in reducing cholesterol absorption in the intestine as proved by increased excretion of untransformed cholesterol, decreased total and non-HDL blood cholesterol, decreased liver cholesterol, and reduction of the atherosclerotic plaque by more than 50% [120]. Since the plant sterols had no effect on gene expression of the different transporters and enzymes involved in intestinal cholesterol absorption, it was concluded and in vitro experienced that plant sterols could displace cholesterol from micelles and therefore inhibit its intestinal absorption [120].

### 8.5. Other Diets

In pigs, high cholesterol intake led to a quadrupled neutral sterols stool excretion, but the ratio of coprostanol to cholesterol in stools was shifted from more than ten to less than one. This provides evidence that the efficiency of cholesterol to coprostanol reduction was limited in this animal model [121]. In parallel, total and LDL blood cholesterol markedly increased when moving from the low to the high cholesterol diet. In the same study, the addition of a cholesterol sequestering agent, β-cyclodextrin, to the hypercholesterolemic diet, brought back cholesterolemia to normal values, while still increasing excretion of neutral sterols in stools, mainly in the form of untransformed cholesterol [121]. Limited bioreduction of cholesterol to coprostanol when dietary cholesterol supply increased was also observed in rats [122]. At last, in three healthy male volunteers, exclusive consumption of a liquid diet for 10 days was accompanied by a drastic drop in fecal excretion of neutral steroids and conversion of cholesterol to coprostanol [65].

Clearly, many, if not all, dietary ingredients have an impact on the biotransformation of cholesterol in the hindgut, but mechanisms are far from all being elucidated. In particular, these changes in cholesterol biotransformation have never been put in perspective with structural and functional modulations of the gut microbiota.

## 9. Coprostanol Formation and Links to Health and Disease

The first associations between cholesterol-to-coprostanol conversion and diseases have relied on gut pathologies. Indeed, it has been shown that the fecal levels of cholesterol and its metabolites were higher in patients with intestinal cancers, adenomatous polyps, and ulcerative colitis [123,124,125]. Moreover, the enzymatic cholesterol-reductase activity was found higher in the stools of colon cancer patients, compared to individuals not suffering from intestinal pathology [126]. Finally, the fecal concentration of coprostanol was significantly higher in patients with colon cancer relative to healthy controls or patients with colon polyps [127]. Altogether, these studies suggest that microbial metabolites of cholesterol could act as co-carcinogens and may increase colon cancer risk. It is not clear whether this association has not been confirmed during the last decades, because of its weak significance or because no dedicated studies were performed recently.

Besides this potential deleterious effect of gut microbial cholesterol metabolism, the impact of gut microbial cholesterol metabolism on host cholesterol homeostasis has been more extensively studied. Gut microbiota contributes to a substantial proportion of the variation in blood lipids, independent of age, gender, and host genetics [102], and an impact of gut microbiota on blood cholesterol has been particularly evidenced. Indeed, germ-free mice have an altered cholesterol metabolism [128], and a recent study showed that microbiota depletion using antibiotics leads to increased blood cholesterol in ApoE-deficient mice. Strikingly, transplant of the microbiota from humans harboring elevated cholesterolemia induced high plasma cholesterol levels in recipient mice [129]. A mathematical model of cholesterol metabolism in the human body, including the gut microbiota, recently revealed that both bile salt metabolism and cholesterol-to-coprostanol conversion can influence blood cholesterol level [130]. Indeed, because coprostanol is poorly absorbable and excreted in the feces, it has been proposed that cholesterol-to-coprostanol conversion by the intestinal microbiota could facilitate the elimination of cholesterol from the body and lower cholesterolemia. This hypothesis was originally proposed by Sekimoto and colleagues, who found in 1983 a negative relationship between cholesterol/coprostanol ratio in feces and cholesterol serum concentration [131]. Later on, several studies with animal models were designed to investigate the effect of feeding of *E. coprostanoligenes* on serum cholesterol concentration. However, only one small-sized experiment including six dietary-induced hypercholesterolemic rabbits, which were daily force fed with live (three rabbits) versus heat-inactivated (three rabbits) *E. coprostanoligenes*, supported a hypocholesterolemic effect of the coprostanoligenic bacterium, with the concomitant increase of coprostanol-to-cholesterol conversion in the proximal digestive tract of the rabbits treated with live bacteria, even after discontinuing the gavage [9]. Other attempts to reproduce this effect in normocholesterolemic lawing hens [8] or germ-free mice [4] daily gavaged with *E. coprostanoligenes*, or alternatively normocholesterolemic germ-free rats fed with *Eubacterium* ATCC 21,408 [56] were unsuccessful, despite the evidence of effective cholesterol-to-coprostanol conversion by the administered active bacteria in all studies. This might suggest that one or two conditions need to be reached for the hypocholesterolemic potential of administered cholesterol-reducing bacteria to be effective in vivo: (i) an hypercholesterolemic condition must exist, and/or (ii) the administered microorganism must be present and active in the proximal digestive tract, where cholesterol absorption takes place. Nevertheless, the hypothesis of an impact of cholesterol-to-coprostanol conversion on blood cholesterol recently emerged again. Indeed, a recent study in overweight postmenopausal women showed an inverse relationship between plasma cholesterol concentrations and fecal coprostanol/cholesterol ratio after nutritional intervention using milk polar lipids [132]. Moreover, the discovery of *ismA* genes described above revealed their potential impact on host cholesterol metabolism. The presence of these *ismA* genes in the microbiome was associated with the presence of coprostanol in stools and lower fecal cholesterol levels. More importantly, the presence of *ismA* genes in human metagenomes was associated with a decrease in total serum cholesterol concentrations, at levels similar to variants in human genes involved in lipid homoeostasis [74]. Finally, relative abundance of IsmA-encoding bacterial species was significantly depressed in Crohn’s disease but not in ulcerative colitis compared to non-inflammatory status. Clearly, these innovative data provide evidence that these 3β-HSDs participate in the first step, and potentially in the last step of cholesterol-to-coprostanol conversion by the human gut and that their abundance correlates with some health conditions, notably, intestinal inflammation and cholesterolemia.

## 10. Conclusions

Despite the fact that cholesterol-to-coprostanol metabolism by the gut microbiota has been known for decades, many questions are still unanswered. Only a few cholesterol-reducing bacteria have been isolated and characterized, and the real diversity of active bacteria is still unknown. Moreover, the real impact of this metabolism on health and disease has not been extensively assessed, and it is even unclear whether it is mainly beneficial or deleterious for human health. The recent discovery of genes implicated in this metabolism and the association of their presence in the gut microbiome with lower blood cholesterol should lead to new research dedicated to the understanding of its influence on disease risks. This could pave the way for the use of the cholesterol-to-coprostanol metabolism as a predictive biomarker of health status and may lead to microbiota-targeted therapeutic interventions, for example, in the context of cardiovascular disease.

## Figures and Tables

**Table 1:** Isolated active strains.

Strains	Isolated from	No. Strains Tested	No. Strains Able to Reduce Cholesterol to Coprostanol	Reference	Comment
*E. coli* *Streptococcus faecalis*^1^ *Clostridium* spp. *Bacteroides* spp. *Bifidobacterium* spp.	Human feces	20 20 20 18 12	0 0 9 12 9	[65]	No strain characterization Not pursued Stains lost
*Eubacterium* 403	Baboon feces	_	One isolate	[69]	Confirmed (59) Strain lost
*Eubacterium* ATCC 21408	Rat caecal contents	_	One isolate	[36]	Confirmed (59) Strain lost
Similar to *Eubacterium* 21408	Feces and intestinal contents of baboons	_	Nine isolates	[57]	Not pursued Strains lost
*Eubacterium* ATCC 51222	Hog sewage lagoon	_	One isolate	[70]	Confirmed Available at ATCC
*Bacteroides* sp. strain D8	Human donor	_	One isolate	[71]	Confirmed Available upon request in our laboratory
*Lactobacilli* *L. acidophilus* ATCC 314 *L. acidophlus* FTCC 0291 *L. bulgaricus* FTCC 0411 *L. bulgaricus* FTDC 1311 *L. casei* ATCC 393	Originally isolated from the human gastrointestinal tract	15	5	[73]	Not pursued Not reproduced Available at ATCC and the Culture Collection Center of Universiti Sains Malaysia (Penang, Malaysia)

^1^ new name: Enterococcus faecalis.
